# Retinal and choroidal changes in steroid-associated central serous chorioretinopathy

**DOI:** 10.1186/s40942-018-0115-1

**Published:** 2018-04-02

**Authors:** Vikas Ambiya, Abhilash Goud, Mohammed Abdul Rasheed, Sankeert Gangakhedkar, Kiran Kumar Vupparaboina, Jay Chhablani

**Affiliations:** 1 Base Hospital, Delhi Cantonment, New Delhi, 110010 India; 2 Srimati Kannuri Santhamma Centre for Vitreo Retinal Diseases, L V Prasad Eye Institute, Kallam Anji Reddy Campus, L V Prasad Marg, Banjara Hills, Hyderabad, 500034 India

**Keywords:** Choroid, CSC, Central serous chorioretinopathy, Steroid-associated CSC

## Abstract

**Background:**

To evaluate the retinal and choroidal alterations in steroid-associated central serous chorioretinopathy (CSC) in comparison to idiopathic CSC.

**Methods:**

In this retrospective cohort study, swept source optical coherence tomography scans of eyes with steroid-associated CSC (group A) were compared with the same in idiopathic CSC (group B). The key features included central subfield retinal thickness, subfoveal choroidal thickness, subfoveal large choroidal vessel diameter, subretinal deposits, retinal pigment epithelial irregularities, double layer sign, hyperreflective dots, intraretinal fluid, and choroidal vascularity index (ratio of choroidal luminal area and total choroidal area, measured on a high definition horizontal 9 mm OCT B-scan.

**Results:**

There were 20 eyes in group A and 30 in group B. Group A had a higher female proportion (60 vs. 16.67%; *P *< 0.01) and higher bilaterality (30 vs. 6.67%; *P *= 0.03). The height of neurosensory detachment was lower in group A (153.1 ± 175.70 µm vs. 312.9 ± 223.06 µm; *P *< 0.01). There was no significant difference in the prevalence of subretinal deposits, retinal pigment epithelial irregularities, pigment epithelial detachments, double layer sign, outer retinal layer disruption, and intraretinal fluid. Hyperreflective dots (HRDs) were less common in group A (15 vs. 46.67%; *P *= 0.03). The subfoveal choroidal thickness (*P *= 0.65) and subfoveal large choroidal vessel diameter (*P *= 0.78) were comparable. There was a trend towards a higher choroidal vascularity index (CVI) in group A (A: mean, 82%, 95% CI, 66–99%; B: mean, 58%, 95% CI, 57–59%; *P *= 0.10).

**Conclusion:**

Steroid-associated CSC has a marginally higher CVI and less common association with HRDs as compared to idiopathic CSC.

## Background

Central serous chorioretinopathy (CSC) is a common chorioretinal disorder, characterized by serous retinal detachment in the posterior pole, often associated with serous pigment epithelial detachments (PED) and retinal pigment epithelium (RPE) atrophy. There is abundant literature supporting the association of CSC with endogenous as well as exogenous hypercortisolism [1–3]. The common exogenous routes of administration of steroids associated with CSC include oral, inhaled, epidural, intra-articular and topical skin ointments [4]. However, the exact role of corticosteroids in the pathogenesis of CSC is not fully understood.

Unlike the idiopathic CSC having a male predominance as high as 8–10:1 [5, 6], steroid associated CSC shows a comparatively lesser predilection for males with a male: female ratio of approximately 3:1 [2, 7–9]. Corticosteroid-induced CSC is also more likely to be bilateral and atypical than idiopathic CSC [10]. Atypical forms of CSC that have been associated with the use of corticosteroids include chronic CSC or diffuse retinal pigment epitheliopathy [9–11], acute bullous retinal detachment [3, 11–13], serous detachment with presence of subretinal fibrin or exudates and subretinal fibrosis [11, 13–17], and bilateral multifocal RPE detachments [11, 18–20].

It has been postulated that steroids cause inhibition of collagen synthase, increased permeability of choroidal capillaries, and dysfunction of ionic pump in the retinal pigment epithelium [21, 22] leading to the accumulation of subretinal fluid. Corticosteroids are known to stimulate release of catecholamines and also to potentiate their effects, which could potentially cause microcirculatory changes in the choroidal vasculature leading to CSC [23]. Glucocorticoids are also known to increase platelet aggregation, thereby causing hypercoagulability, increased microthrombus formation, increased blood viscosity, all of which could affect the choroidal microcirculation [24, 25].

The main proposed pathomechanism for CSC is hyperpermeability of choroidal vessels. ICGA in eyes with CSC demonstrates choroidal vascular abnormalities in the form of delay in choroidal filling, abnormally dilated choroidal vasculature in the early phase and choroidal hyperpermeability in the late phase [26, 27]. Laser doppler flowmetry has shown decreased foveal choroidal blood flow [27], whereas laser interferometry has shown choroidal hyperperfusion in CSC patients [28], suggesting that the blood flow might be variable in different layers of the choroid. By using enhanced depth imaging- optical coherence tomography (EDI-OCT), it has been observed that subfoveal choroidal thickness (SFCT) is increased in eyes with CSC as compared to normal eyes [29–31] In addition, we reported that in patients with acute CSC, “choroidal vascularity index” (CVI), a ratio of luminal area and total choroidal area, is significantly higher as compared to the fellow eyes and also normal age-matched controls [32].

In view of the various postulated effects of corticosteroids on the choroidal vasculature, and the role of choroidal vascular perfusion in the pathogenesis of CSC, it would be interesting to understand the changes in choroid especially CVI in steroids-associated CSC. We compared eyes with steroid-induced CSC, with those having idiopathic CSC without any history of exposure to steroids. We intended to look for differences in the clinical presentation and the optical coherence tomography (OCT) features between the two groups, especially choroidal changes.

## Methods

A retrospective chart analysis of eyes with a diagnosis of CSC was done at a tertiary eye care centre, to compare those cases with a history of steroid exposure (Group A) versus those with no history of steroid exposure in the past (group B). The study duration was from January 2015 to July 2017.

The inclusion criteria were: (1) age ≥ 18 years; (2) acute or chronic CSC diagnosed by the presence of subretinal fluid at fovea, verified by OCT; (3) Group A: who were exposed to corticosteroids within 12 months prior to the development of CSC; Group B: who did not have a history of current or prior exposure to any type of corticosteroids.

The exclusion criteria were: (1) any past intervention in the form of laser photocoagulation/photodynamic therapy (PDT)/vitreoretinal surgery/intravitreal injection/oral therapy for CSC; (2) vitreoretinal/macular disorders other than CSC currently or in the past (3) evidence of glaucoma (4) spherical equivalent ≥ ± 6 D (5) cataract surgery in the past 6 months (6) any media opacity likely to cause attenuation of signal strength in OCT (7) history of malignant hypertension (8) pregnancy.

A detailed ocular history (onset of symptoms, previous treatment), the demography (age, gender), laterality, systemic comorbidities (diabetes and hypertension) were recorded. A detailed history of the duration and route of steroid exposure was taken. Institutional review board approval was obtained for retrospective data collection and analysis.

The clinical examination included assessment of the best corrected visual acuity (BCVA) in Snellen, spherical equivalent of refractive status of the eye, slit lamp biomicroscopy with a contact lens or non-contact lens, indirect ophthalmoscopy, and digital fundus fluorescein angiography (FFA) and ICGA as per physician discretion. All eyes underwent swept–source OCT (SS-OCT) imaging on “Triton” (Topcon Corporation, Tokyo, Japan) to obtain central subfield retinal thickness (CMT), and the SFCT. The SFCT was measured at subfoveal location as the vertical distance between the hyperreflective line of Bruch’s membrane and the innermost hyperreflective line of the chorio–scleral interface. Subfoveal large choroidal vessel diameter (SLCVD) was measured as the vertical distance from the top of the large choroidal vessel in the outer choroid within central 500 microns. Other features noted in OCT included height of neurosensory detachment (NSD), presence of subretinal deposits, RPE irregularities, PED, double layer sign (defined as irregular shallow PEDs with hyper-reflective content inner to an intact hyper-reflective Bruch membrane), outer retinal layer disruption, hyperreflective dots (HRDs), intraretinal fluid, CVI. All OCT scans were performed between 9:00 am and 12:00 pm. The various clinical and tomographic features were compared between the two groups.

CVI calculation: The CVI calculation was done using previously reported algorithm [33]. Briefly, choroidal stroma and vessel area analysis involved (1) automated binarization of a high definition horizontal 9 mm OCT B-scan and (2) automated segmentation of the binarized choroid layer as reported previously. The task of automated binarization in turn involved (a) preprocessing, (b) exponential and non-linear enhancement, and (c) thresholding.

### Statistical analysis

The Snellen BCVA was converted to logarithm of the minimum angle of resolution (logMAR) equivalent for statistical analysis. The numerical variables between groups A and B were compared using Mann–Whitney *U* Test. The association of categorical variables with the two groups was calculated in the form of odd ratio, and the significance analysed using Fisher’s exact test. *P* value of < 0.05 was considered as statistically significant.

## Results

**Fig. 1:**
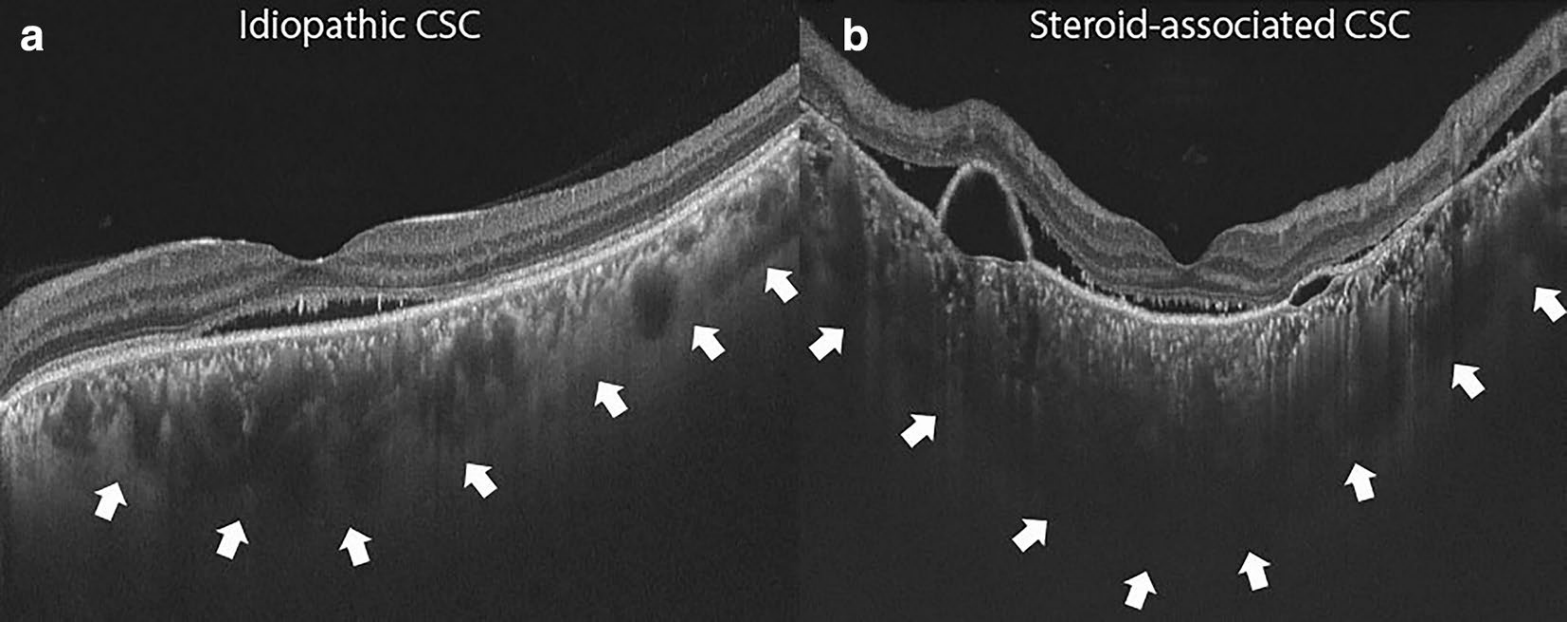
**a** Swept source optical coherence tomography (SS-OCT) scan of a 40 years old male, with a diagnosis of idiopathic central serous chorioretinopathy (CSC), with best corrected visual acuity (BCVA) of 20/20. **b** The SS-OCT scan of a 43 years old male, with steroid-associated CSC, with BCVA of 20/40, and a history of exposure to oral steroids for the past 1 month, showing a thicker choroid, and more hyperreflective foci in comparison to **a**

The study included 20 eyes of 16 patients in group A (steroid associated CSC) and 30 eyes of 26 patients in group B (no history of exposure to steroids). Of the 16 patients in group A, there was history of exposure to oral steroids in ten (62.5%) cases, parenteral steroids in two (12.5%) cases, inhalational steroids in three (18.75%) cases, and topical steroids in one (6.25%) case. Representative cases of both groups are shown as Fig. 1.

The baseline features of the two groups are summarised in Table 1. The baseline features in both groups were comparable in terms of the mean age (group A: 45 ± 16.13 years; group B: 43.3 ± 9.45 years; *P *= 0.77), mean duration of symptoms (group A: mean, 105.83 days, range, 4–300 days; group B: mean, 107.13 days, range, 10–365 days; *P *= 0.98), and the mean baseline best corrected visual acuity (group A: 20/33 Snellen ± 0.34 logMAR; group B: 20/40 Snellen ± 0.27 logMAR; *P *= 0.08). However, there was a significantly higher female proportion of cases in group A as compared to B (group A: 60%, 12 of 20 eyes; group B: 16.67%, 5 of 30 eyes; *P *< 0.01). Bilaterality of CSC was significantly commoner in the steroid group (group A: 30%, 6 of 20 eyes; group B: 6.67%, 2 of 30 eyes; *P *= 0.03).

**Table 1:** Baseline features of eyes with steroid associated CSC (group A) and those with idiopathic CSC (group B)

Baseline features	Group A (N = 20 eyes of 16 patients)	Group A (N = 30 eyes of 28 patients)	*P* value
Age (years ± SD)	45 ± 16.13	43.3 ± 9.45	0.77
Male: female (n)	12:8	5:25	< 0.01*
Bilaterality	6/20 (30%)	2/30 (6.67%)	0.03*
Duration of symptoms (days ± SD)	105.83 ± 96.10	107.13 ± 138.70	0.98
Baseline BCVA (Snellen ± SD logMAR)	20/33 ± 0.34	20/40 ± 0.27	0.06

*CSC* central serous chorioretinopathy; *SD* standard deviation; *BCVA* best corrected visual acuity

* Statistically significant

**Fig. 2:**
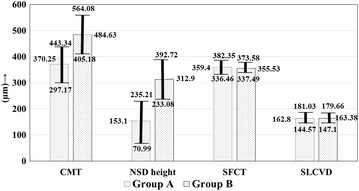
Figure shows the comparison of the quantitative optical coherence tomography (OCT) measurements in eyes with steroid associated serous chorioretinopathy (CSC) (group A) versus those having idiopathic CSC (group B) in mean (µm) ± 95% CI. The central subfield retinal thickness (CMT) is marginally lower whereas the height of neurosensory detachment (NSD) is significantly lower in group A. The subfoveal choroidal thickness (SFCT) and subfoveal large choroidal vessel diameter (SLCVD) are comparable

**Fig. 3:**
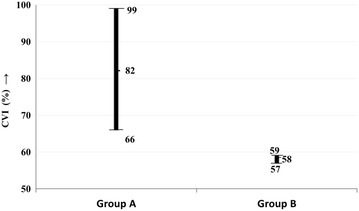
Figure shows the comparison of the choroidal vascularity index (CVI) in mean ± 95% CI, between eyes with steroid associated serous chorioretinopathy (CSC) (group A) versus those having idiopathic CSC (group B). CVI is a measure of the ratio of choroidal luminal content to the choroidal stromal content, and is calculated from swept source optical coherence tomography (SS-OCT) imaging. The CVI is higher in group A indicating higher choroidal vascularity in steroid associated CSC

The OCT features of the two groups are summarized in Table 2. The CMT was lower in group A (Group A: mean, 370.25 µm, 95% CI, 297.17–443.34 µm; Group B: mean, 484.63 µm, 95% CI, 405.183–64.08 µm; *P *= 0.08), though not statistically significant (Fig. 2). The NSD height was however significantly lower in group A (Fig. 2) as compared to B (group A: 153.1 ± 175.70 µm; group B: 312.9 ± 223.06 µm; *P *< 0.01). Between the two groups, there was no significant difference in the proportion of cases having subretinal deposits (*P *= 0.22), RPE irregularities (*P *= 0.49) and PEDs (*P *= 0.54). Similarly there was no significant difference in the proportion of cases having double layer sign (*P *= 0.35), outer retinal layer disruption (*P *= 0.51), and IRF (*P *= 0.51). However there was a significantly lower proportion of eyes in group A having HRDs on OCT (group A: 3 of 20, 15% eyes; group B: 14 of 30, 46.67% eyes; *P *= 0.03; Odds ratio, 0.20, 95% CI, 0.05–0.84; *P *= 0.03). The chorioscleral interface was visible in all cases. There was no significant difference in the SFCT (*P *= 0.65) and SLCVD (*P *= 0.78) between the two groups (Fig. 2). There was a trend towards a higher CVI in group A (group A: mean, 82%, 95% CI, 66–99%; group B: mean, 58%, 95% CI, 57–59%; *P *= 0.10) (Fig. 3).

**Table 2:** OCT features of eyes with steroid induced CSC (group A) and those with idiopathic CSC (group B)

	Group A N = 20 eyes	Group B N = 30 eyes	*P* value	OR (95% CI)	*P* value
CMT (µm ± SD)	370.25 ± 156.39	484.63 ± 222.02	0.08	–	–
NSD height (µm ± SD)	153.1 ± 175.70	312.9 ± 223.06	< 0.01*	–	–
Subretinal deposits	4/20 (20%)	12/30 (40%)	0.22	0.38 (0.10–1.40)	0.14
RPE irregularities	5/20 (25%)	5/30 (16.67%)	0.49	1.67 (0.41–6.73)	0.47
PED	5/20 (25%)	11/30 (36.67%)	0.54	0.58 (0.16–2.02)	0.39
Double layer sign	4/20 (20%)	11/30 (36.67%)	0.35	0.43 (0.11–1.62)	1.24
Outer retinal layer disruption	0/20 (0%, 95% CI, 0–16.11%)	2/30 (6.67%)	0.51	0.28 (0.01–6.10)	0.42
Hyperreflective dots	3/20 (15%)	14/30 (46.67%)	0.03*	0.20 (0.05–0.84)	0.03*
Intraretinal fluid	0/20 (0%, 95% CI, 0–16.11%)	2/30 (6.67%)	0.51	0.28 (0.01–6.10)	0.42
SFCT (µm ± SD)	359.4 ± 49.10	355.53 ± 50.42	0.65	–	–
SLCVD (µm ± SD)	162.8 ± 39.01	163.38 ± 49.26	0.78	–	–
CVI	82 ± 35%	58 ± 3%	0.10	–	–

*OCT* optical coherence tomography; *CMT* central macular thickness; *NSD* neurosensory detachment; *RPE* retinal pigment epithelium; *PED* pigment epithelial detachment; *CT* choroidal thickness; *SLCVD* subfoveal large choroidal vessel diameter; *CVI* choroidal vascularity index; *OR* odds ratio; *CI* confidence interval

* Statistically significant

## Discussion

Our study shows that steroid-induced CSC more commonly has bilateral presentation than the idiopathic form, which is in agreement with previous reports [10, 34]. Similarly we found female preponderance in the former as opposed to the male preponderance known in the idiopathic form [10, 34].

HRDs are seen in CSC in the subretinal space below the NSD [35, 36] and within the retinal layers [37, 38]. Intraretinal precipitates in CSC may result from the accumulation of proteins or macrophages with the phagocytized photoreceptor outer segments. These HRDs are more frequently observed in the chronic and recurrent forms of CSC [39]. We found significantly less HRDs in group A. However, as the duration of symptoms was comparable between the two groups, we cannot attribute this difference to chronicity in either group. We postulate that exposure to steroids might be associated with lesser accumulation of proteins and decreased phagocytosis of outer segments by macrophages, leading to less number of HRDs.

The presence of “double layer sign” on OCT was comparable in both groups. This sign is more commonly seen in chronic CSC [40, 41]. Other signs of chronicity, namely RPE irregularities, IRF, and outer retinal degeneration were also comparable in the two groups.

The present study found a higher CMT and a higher NSD in idiopathic CSC as compared to steroid associated CSC. However, there was no significant difference in the SFCT and the SLCVD. In contrast, we found a definite trend towards a higher CVI in the steroid group suggestive of a higher choroidal vascular component compared to the choroidal stromal component in those eyes. Yang et al. [41], in their study on the choroidal vascular changes in CSC, have reported that the largest diameter of choroidal hyporeflective lumen in eyes with CSC, is not significantly enlarged compared with the fellow eye. We propose that CVI could be considered a better indicator of choroidal vascularity as compared to large choroidal vessel diameter proposed by Yang et al. [41], as the latter is presumed on a single B scan, whereas a better assessment of the actual diameter of the choroidal vessels could only be accomplished with 3D modelling of the choroid, rather than measuring one vessel at one point.

Although the association of corticosteroids with CSC is well established, we found only one recent study comparing the choroidal features of steroid-associated CSC with those of idiopathic CSC [42]. The authors found that eyes with steroid-associated CSC have a thinner central choroid, lesser choroidal vessel dilatation, and a lesser choroidal vessel hyperpermeability as compared to eyes with idiopathic CSC. Further to this, even the fellow eyes in case of the steroid-associated CSC had a thinner central choroid as compared to those in case of idiopathic CSC. Given that the pathomechanism of idiopathic CSC is related to increased choroidal thickness, choroidal vessel dilatation and hyperpermeability [43, 44], the authors concluded that secondary CSC should be caused by a different pathomechanism, which does not manifest with any of these features. However, the authors have assessed the choroidal vessel dilatation and hyperpermeability from the cyanescence on ICGA, which cannot accurately quantify these parameters. Contrary to their findings, we found CVI to be higher in steroid associated CSC, which indicates a higher choroidal vascularity in these cases. Yet again, CVI should be considered a better assessment of choroidal vascularity than that assessed from the vascular diameter and late leakage seen on a two-dimensional image of ICGA.

Studies on rodents have shown that glucocorticoids act on mineralocorticoid receptors in the choroidal vascular endothelium, thereby causing choroidal vascular leakage and dilatation by upregulating the endothelial vasodilatory K channel KCa2.3. [45]. We hypothesize that the preferential vascular dilatation manifests in the form of increased CVI in steroid associated CSC.

Our study has the inherent limitations of a retrospective study and the fewer number of eyes studied can also not be overlooked. The unmatched sex ratio between the two groups could have caused bias, as the association of sex with CT has been reported [46]. Moreover, the systemic conditions for which the cases in group A received steroids, might also have independently affected the OCT features. It also is important to note that OCT does not evaluate the permeability of vessels and the vascular dilation seen on a B scan is an indirect measurement of the permeability or inflammation.

## Conclusion

In conclusion, steroid associated CSC has higher CVI, and a lower prevalence of HRDs on SS-OCT in comparison to idiopathic CSC. However, changes in these anatomical parameters during the follow up will expand our understanding about the role of steroids in association with CSC.
